# Smart Protocols for Physical Therapy of Foot Drop Based on Functional Electrical Stimulation: A Case Study

**DOI:** 10.3390/healthcare9050502

**Published:** 2021-04-26

**Authors:** Jovana Malešević, Ljubica Konstantinović, Goran Bijelić, Nebojša Malešević

**Affiliations:** 1 Tecnalia Serbia Ltd., 11000 Belgrade, Serbia; 2 Faculty of Medicine, University of Belgrade, 11000 Belgrade, Serbia; ljubica.konstantinovic@mfub.bg.ac.rs; 3 Clinic for Rehabilitation “Dr. Miroslav Zotović”, 11000 Belgrade, Serbia; 4 Tecnalia, Basque Research and Technology Alliance (BRTA), 20009 Donostia-San Sebastián, Spain; goran.bijelic@tecnalia.com; 5 Department of Biomedical Engineering, Faculty of Engineering, Lund University, 22100 Lund, Sweden

**Keywords:** functional electrical stimulation, foot drop, smart protocols, physical therapy, contralateral control, range of motion, stroke

## Abstract

Functional electrical stimulation (FES) is used for treating foot drop by delivering electrical pulses to the anterior tibialis muscle during the swing phase of gait. This treatment requires that a patient can walk, which is mostly possible in the later phases of rehabilitation. In the early phase of recovery, the therapy conventionally consists of stretching exercises, and less commonly of FES delivered cyclically. Nevertheless, both approaches minimize patient engagement, which is inconsistent with recent findings that the full rehabilitation potential could be achieved by an active psycho-physical engagement of the patient during physical therapy. Following this notion, we proposed smart protocols whereby the patient sits and ankle movements are FES-induced by self-control. In six smart protocols, movements of the paretic ankle were governed by the non-paretic ankle with different control strategies, while in the seventh voluntary movements of the paretic ankle were used for stimulation triggering. One stroke survivor in the acute phase of recovery participated in the study. During the therapy, the patient’s voluntary ankle range of motion increased and reached the value of normal gait after 15 sessions. Statistical analysis did not reveal the differences between the protocols in FES-induced movements.

## 1. Introduction

Functional electrical stimulations (FES) has been used for correcting foot drop for more than half a century. People with foot drop have reduced active control of the foot due to a combination of weak dorsiflexors and increased spasticity and stiffness of the plantar flexors [1]. Most of the current available FES systems stimulate the tibialis anterior to induce dorsiflexion (DF) during the swing phase of gait to ensure foot clearance [2,3,4]. Employment of the FES during walking proved to be not only assistive but also effective as a therapeutic device. Increases in walking speed, stride/step length, foot range of motion (RoM), functional mobility, and reducing spasticity are some of the presented benefits of FES-assisted walking [3,5,6,7,8,9,10]. Studies have shown that stroke survivors, besides compromised DF, do not effectively activate their plantar flexors during gait [11,12]. Plantar flexors have an important role in forward propulsion, swing initiation and power generation, and provide the majority of the stability for the entire body during the mid-stance phase of the gait [13,14].

Interruptions in the blood supply to the brain during a stroke lead to the loss of neurons that had highly specific functions. Reorganization in the damaged hemisphere leads to better rehabilitation. Studies suggested that specific training and rehabilitation programs that target the use of the hemiparetic limbs, such as repetitive and intense movement practice, an active engagement of the patient during therapy, and functional gains following task-specific training, can improve neuroplasticity [15,16,17,18] and consequently improve motor control. Additionally, neural plasticity could be promoted by bilateral movements that allow the activation of the undamaged hemisphere to increase activation of the damaged hemisphere and facilitate control of the paretic limb [19].

Burgar et al. [20] presented a robot for clinical therapy that continuously moved the paretic limb to the mirror-image position of the healthy limb enabling the patient to practice bimanual shoulder and elbow movements in the horizontal plane. Clinical trials comparing bilateral robot therapy to traditional therapy in 21 moderately affected chronic stroke subjects showed significant improvement in the strength of the biceps, triceps, and deltoideus muscles, and in the elbow and shoulder section of the Fugl-Meyer movement assessment score. Whital et al. [21] introduced a repetitive bilateral arm training with rhythmic cueing protocol, which consisted of moving two handles in a reaching motion symmetrically (arms moving together in the same direction) and asymmetrically (one arm pushes away while the other pulls towards). They showed improvements in Fugl-Meyer scores and the Wolf Motor function test. The range of motion and said improvements were maintained at two months after patients stopped training.

Recent studies used FES to stimulate paretic hand depending on the movement of the healthy hand. Knutson et al. described the new treatment that uses contralaterally controlled functional electrical stimulation (CCFES) [22]. Up to three surface electrodes were placed over the forearm finger and thumb extensors to produce functional hand opening. The healthy hand wore the command glove with sensors attached to the dorsal side. The glove detected the degree of hand opening, and based on that, the strength of muscle contraction was modulated with pulse duration. The results from both chronic and subacute patients showed greater improvements in CCFES groups compared with the groups that had cyclic neuromuscular electrical stimulation [22,23,24]. Another CCFES study included patients six weeks after onset of stroke [25].

Inspired by promising results of CCFES for the upper limbs, Knutson et al. developed a new CCFES system for the lower limbs [26]. The DF of the paretic ankle was controlled by volitional dorsiflexion of the unaffected ankle. The movement of the unaffected ankle was tracked with a bend sensor placed on a sock. The CCFES therapy was compared with the cyclic FES, where stimulation was activated according to the established scheme. All patients that participated were in the chronic phase. Patients from both groups showed significant changes across all the tested impairment measures and the functional ambulation measure, but there was no significant benefit of CCFES compared to the cyclic FES. Authors suggested that patients in the acute phase could benefit more from this type of therapy and also that the pattern of stimulation activation should correspond to the pattern of walking, i.e., the DF of one foot should be synchronized with the plantar flexion (PF) of the other.

This pilot study aimed to propose a novel rehabilitation program for treating foot drop in patients who could not use FES when walking because of at least one of the following conditions: disturbed balance, insufficient physical fitness, a lack of locomotor control, spasticity, etc. This program integrated several previously-researched control techniques that might promote and/or speed up motor recovery: (1) FES-induced foot DF and PF movement [27]; (2) repetitive, goal-oriented movement training [22]; (3) active participation of the patient [28,29]; (4) bilateral in-phase and anti-phase movements [19]. The program consisted of a set of seven different protocols, called smart protocols. In six of the seven protocols, movements of the paretic foot were controlled contralaterally by the healthy foot, while in one protocol, the active movements of the paretic foot triggered the stimulation. We analyzed FES-induced movements during individual sessions and during the therapy. Moreover, we tracked the effectiveness of a novel FES therapy through voluntary ankle RoM and the ability to perform the desired foot movement.

## 2. Materials and Methods

### 2.1. Subject

The participant in the study was a 63-year-old female patient. The treatment started 2.5 months after she suffered an ischemic stroke insult. Her left side was affected. The patient could not walk. She needed someone’s assistance to transfer her from a lying to a sitting position and could not stand independently with a tendency to fall to the left. Besides the FES treatment described below, the patient received the conventional stroke rehabilitation program, including 60 min of conventional physiotherapy based on the neurodevelopmental facilitation approach (Bobath concept) and 30 min of daily occupational therapy. The occupational therapy included functional mobility exercises, sitting balance, transferring in/out bed/chair, and wheelchair management. The baseline of the lower extremity Fugl-Meyer Movement Assessment [30] was 13 out of 34, the Barthel Index [31] was 80 out of 85, and the Mini-Mental State Examination [32] 30 out of 30. The experimental procedures and potential risks were explained to the patent and she signed a written consent. Ethical approval for the study was obtained from the local ethics committee. The study was conducted according to the guidelines of the Declaration of Helsinki, and ethical approval was obtained from the Ethics Committee of the Clinic for Rehabilitation “Dr. Miroslav Zotović” affiliated with the Faculty of Medicine, University of Belgrade (the ethics board protocol number: 03-295/1, date of approval: 29 January 2016).

### 2.2. Hardware

Stimulator with electrodes: An electrode with sixteen conductive pads arranged in two rows placed over the lateral and medial popliteal fossa and the IntFES v2 stimulator (Tecnalia R&I, Donostia-San Sebastián, Spain) [33] were used for inducing DF and PF of the paretic foot. The common anode was positioned below the knee. The stimulator delivered a single biphasic pulse train to the demultiplexer, which routed it to different independent conductive pads within the multi-pad electrode. The ARM processor was utilized for the execution of various stimulation protocols, pulse-by-pulse control of the stimulation pattern, real-time acquisition, processing of the data stream from sensors, and communication with a host controller. The control of the stimulation process was done via the National Instruments LabVIEW (National Instruments, Austin, TX, USA) application with a user-friendly graphical interface, running on a tablet PC. The stimulator output stage was current controlled in 1 mA steps with the maximal current limited to 50 mA. The operating principle that supported multi-pad electrode based FES paradigm with increased movement selectivity and delayed onset of muscle fatigue in comparison to conventional FES was reported in our previous studies [33,34].

**Figure 1:**
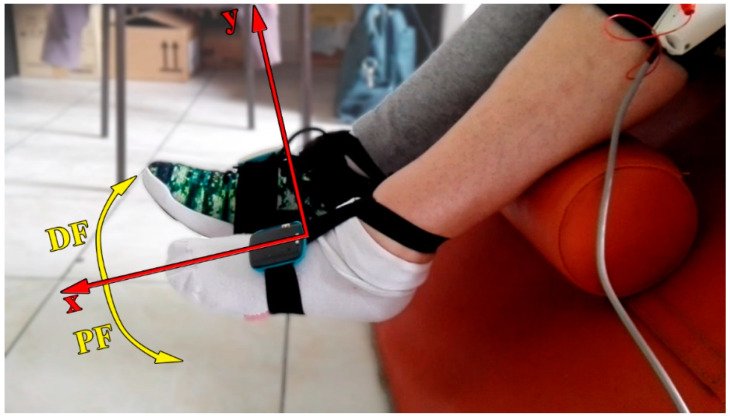
The position of the patient during a session. The patient sat in the wheelchair with both legs hanging over the wedge pillows. Wireless sensors were placed onto the insets of both feet. The paretic foot was without a shoe to minimize any extra weight. The reference 2D coordinate systems were established with zero values in line with each foot sensor when feet were relaxed. Movement of the foot above the zero value was considered as dorsiflexion (DF), and movement of the foot below zero value was considered as plantar flexion (PF).

Assessment: During treatment, two wireless measurement units were placed onto the insets of both patient’s feet. They were attached with a combination of elastic bands and hooks-and-loop straps, allowing secure and easy fastening onto the patient’s foot with minimal displacement. Each of the sensors comprised of a micro-accelerometer and a micro-gyroscope in a single chip (MPU-6050, InvenSense, San Jose, CA, USA) (Figure 1).

### 2.3. Smart Protocols

During the smart protocols, the movement of the paretic foot was FES-induced by: (1) moving the healthy foot (contralaterally controlled) or (2) the residual voluntary movement of the paretic foot. In six of the seven protocols, movement of the paretic foot was controlled contralaterally in-phase or anti-phase in the sagittal plane. Movements were considered as in-phase when DF (PF) of one ankle was accompanied by DF (PF) of the other, and as anti-phase when DF (PF) of one ankle was accompanied by PF (DF) of the other. Although the paretic foot was moved without any voluntary movement (due to FES), the patient was still instructed to try to simultaneously move the paretic foot in the specified direction (the same direction as the direction of FES-induced movement). In the last protocol, stimulation was initiated with voluntary movements of the paretic foot. The order of smart protocols was randomly selected before the first session and the same order remained in all of the sessions.

The patient operated the system by herself. She was able to choose the rate, the direction, and the strength of the movements. In protocols in which the duration of the stimulation was not predefined, the patient could also decide to cease the stimulation (smart protocols 1.1, 1.2, 3.1, and 3.2). The predefined duration of the stimulation in protocols 2.1, 2.2, and 4 was chosen following data from other studies [35,36,37]. The main idea behind protocols 1–3 was to provide different levels of control to the user. During protocol 2 (2.2 and 2.2), the patient controls only direction and initiation. This control is extended in protocol 1 (1.1 and 1.2) by adding the contraction duration as a controllable parameter. Finally, in protocol 3 (3.1 and 3.2) the patient was able to set the stimulation amplitude, resulting in a variable level of muscle contraction. By expanding the controllability of FES protocols in incremental steps, the intention was to explore both the effectiveness and personal preference of the patient in adopting new therapy protocols. The FES was delivered at a frequency of 40 Hz and with a pulse width of 400 μs throughout the whole smart protocols therapy. The current amplitudes selected to elicit DF and PF were adjusted just before the beginning of smart protocols, in accordance with our previous study [38], and were within the same range as previously reported (16–24 mA).

In the figures within Table 1, simultaneous feet movements are marked with the same color. Yellow parts of the arrows on the healthy foot represent the neutral zone, i.e., zone without stimulation.

### 2.4. Treatment

The patient had 15 therapy sessions, once per day, five times per week during weekdays. The therapy was performed at the Clinic for Rehabilitation ˝Dr. Miroslav Zotović˝ in Belgrade, Serbia. Each session was divided into four sections: (1) system setup; (2) recording of the voluntary and passive (performed by the therapist) movement of the paretic foot; (3) creating the pads configurations for DF and PF within multi-pad electrode; (4) smart protocols. The first three steps are explained in detail in our previous publication [38], the difference being the position of the patient. In this study, the patient sat in the wheelchair with both legs over the wedge pillows (Figure 1). This position allowed unobstructed feet movement. Except for producing a quality movement, the activation of final stimulation configurations should not cause a painful or unpleasant sensation. In cases when the patient reported such sensations, the pads included in the configuration or/and stimulation amplitude would be adjusted. Further, the patient could ask for the stimulation parameters to be changed at any time during the session. Pad configurations for DF and PF during each session were stored in stimulator memory. The patient’s paretic foot was without a shoe to avoid shifting extra weight. The shoe on the healthy foot did not cause a significant difference in ankle mobility compared to when the shoe was off. Hence, we decided to keep the shoe on the foot to avoid additional engagement of a clinician, in terms of putting on and taking off the shoe.

Each one of the seven smart protocols lasted for three minutes, with a one-minute pause between two consecutive protocols. The duration of one session (7 × 3 + 6 × 1 = 27 min) was the same as for FES-assisted walking applied in previous studies [39,40].

### 2.5. Assessment

Movements of the foot in the sagittal plane were estimated based on the gravitational component of the acceleration and calculated as the arctangent of the ratio of the acceleration values in transverse and sagittal planes, according to the method described in [41].

The calculation of feet trajectories was independent for each foot. At the beginning of each smart protocol, the patient’s feet were relaxed. These positions were marked as zero values in the 2D systems. Foot movements above zero value were considered as foot DF and movements below zero value as foot PF (Figure 1).

## 3. Results

**Figure 2:**
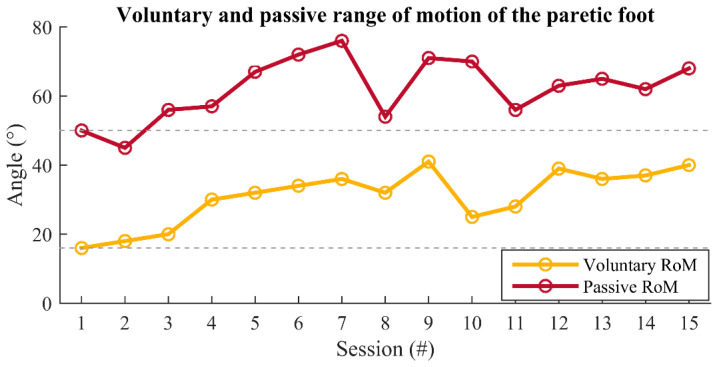
The voluntary (yellow line) and passive (red line) ankle RoMs. Ankle RoMs were recorded at the beginning of each session throughout the therapy.

The participant completed 15 therapy sessions. The voluntary and passive range of motion in the sagittal plane of the paretic foot was recorded at the beginning of each session throughout the therapy, and the results are presented in Figure 2. The voluntary RoM increased from 16° at the baseline to 40° at the end of the therapy, while the passive increased from 50° to 68°.

Figure 2 also shows a serious issue that can be expected during stroke rehabilitation—the increase in the patient’s joint stiffness occurring on some days during the study. As noticeable on days 8, 10, and 11, there were significant drops in passive and/or active RoM, which was not in line with the recovery trend from previous days.

**Figure 3:**
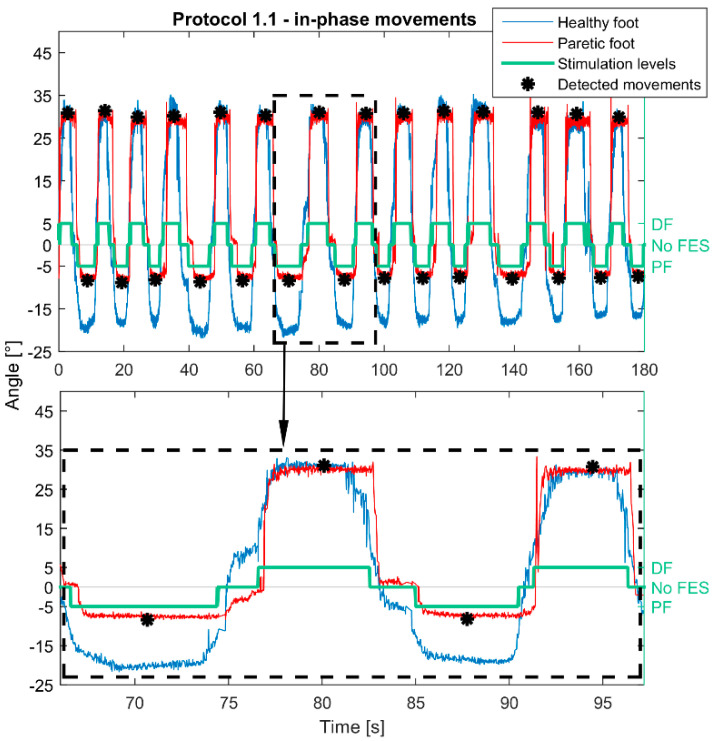
Example of Smart protocol 1.1. Movements were in-phase in the sagittal plane and the paretic foot was controlled by the healthy. Passing by the set thresholds (10°/−10° relive to the coordinate origin established at the beginning of the protocol) of the healthy foot activated configurations for dorsiflexion (DF)/plantar flexion (PF) of the paretic foot. When the healthy foot was between set thresholds, the stimulation was off. Calculated movements of the healthy foot are represented with red, and movements of the healthy foot with the blue line. Stimulation activations are shown with the green line. Estimated positions of the achieved FES-induced movements are marked with black asterisks.

**Figure 4:**
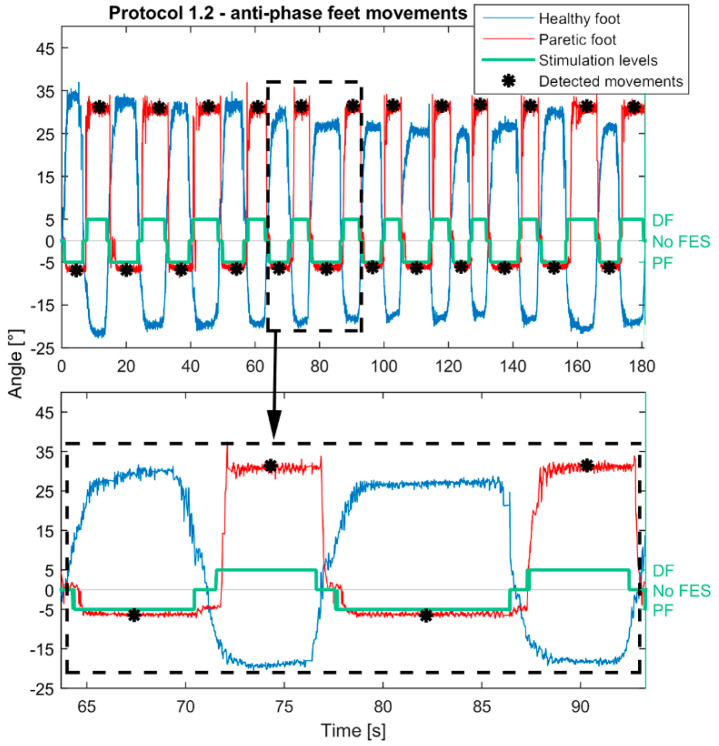
Example of the Smart protocol 1.2. Movements were in anti-phase in the sagittal plane and the paretic foot was controlled by the healthy. Passing by the set thresholds (10°/−10° relive to the coordinate origin established at the beginning of the protocol) of the healthy foot activated configurations for plantar flexion (PF)/dorsiflexion (DF) of the paretic foot. When the healthy foot was between set thresholds, the stimulation was off. Calculated movements of the healthy foot are represented with red, and movements of the healthy foot with the blue line. Stimulation activations are shown with the green line. Estimated positions of the achieved FES-induced movements are marked with black asterisks.

To analyze the effects of smart protocols, each FES-induced movement of the paretic foot was represented by an angle. In protocols 1.1, 1.2, 2.1, and 2.2 the angle was calculated as the median value of the plateaus of an FES-induced movement in the sagittal plane. In protocols 3.1 and 3.2, the movement of the healthy foot activated stimulation patterns with several current intensities depending on the heathy foot position. For these protocols, the angle was calculated as the median value of the highest plateaus (stimulation pattern with the highest current amplitude) between two consecutive states without stimulation. Examples of feet trajectories and how paretic foot movement angles were detected when feet movements were in-phase (protocol 1.1) are shown in Figure 3. Similarly, Figure 4 shows paretic foot angle detections during anti-phase movements (protocol 1.2) in Green lines in Figure 3 and Figure 4 illustrate FES pattern activation: positive values represent activation of DF and negative activation of PF.

**Figure 5:**
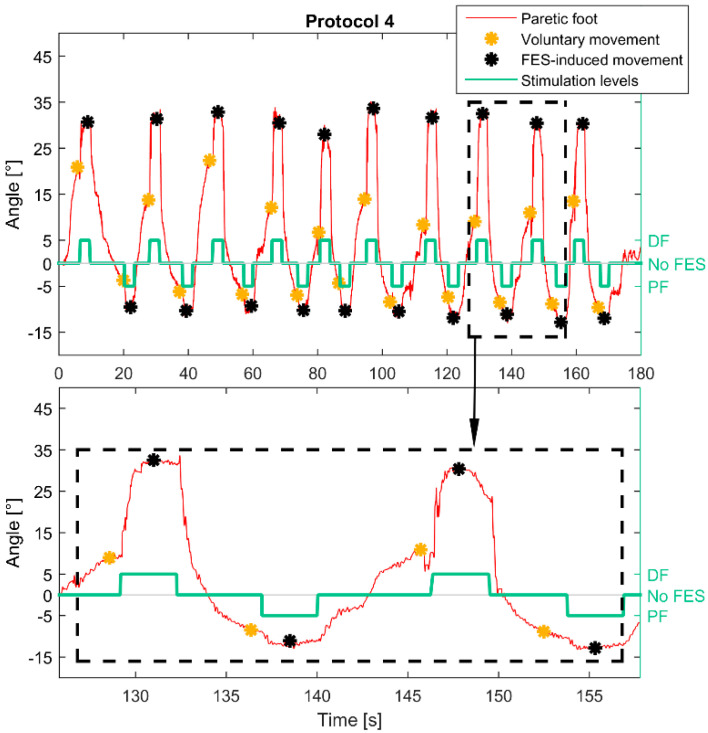
Smart protocol 4. The calculated movements of the paretic foot are presented with the red line. The detected maximal voluntary movements are marked with yellow asterisks. The stimulation was activated a half second after the detected maximal voluntary movement. The duration of FES-induced movement was three seconds and its estimated value is marked with the black asterisk.

In smart protocol 4, movements were induced by the paretic foot. The stimulation pattern was activated when there was not increase/decrease in movement amplitude for 0.5 s. The angle 0.5 s before FES activation represents voluntary movement and median value from the activation to the end of stimulation FES-induced movement. Figure 5 shows an example of the paretic foot trajectory, detected representative voluntary, and FES-induced movement angles during smart protocol 4.

**Figure 6:**
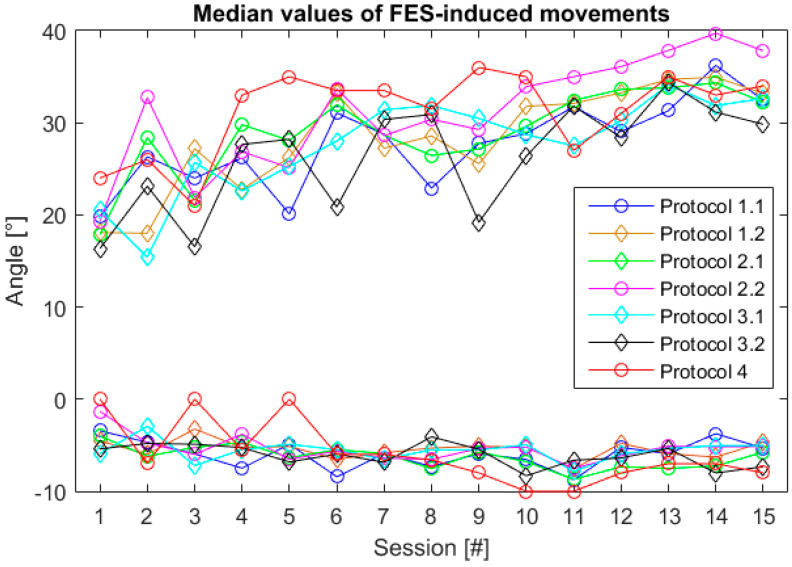
Session’s median values of representative voluntary and FES-induced dorsiflexion (positive values) and plantar flexion (negative values) angles throughout 15 therapy sessions for all Smart protocols.

The session’s median values of representative angles for all Smart protocols throughout the therapy are shown in Figure 6. Positive values correspond to FES-induced DF and negative to FES-induced PF movements.

**Figure 7:**
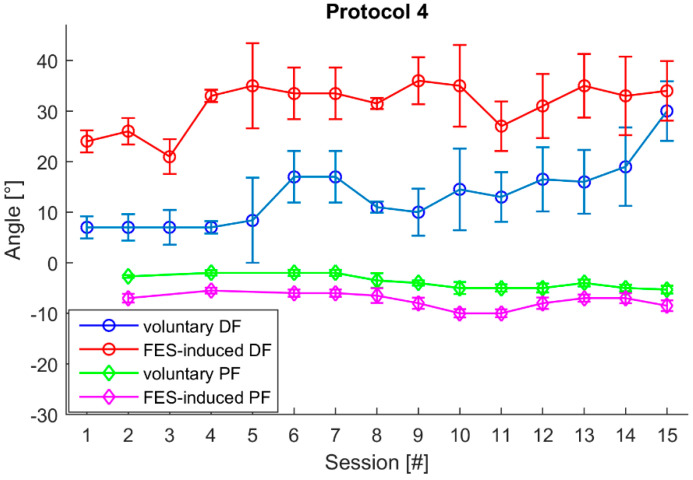
The calculated session’s median values of representative voluntary and FES-induced dorsiflexion (positive values) and plantar flexion (negative values) during Smart protocol 4 throughout 15 therapy sessions.

The session’s median values of representative voluntary and FES-induced angles for Smart protocol 4 are shown in Figure 7. In sessions 1, 3, and 5 the patient did not succeed in performing satisfactory PF movement, i.e., in lowering the paretic foot 2° from the coordinate origin established at the beginning of the protocol. After eight sessions, the patient managed to perform any desired movement.

## 4. Discussion

Due to the fact that the conservation of cortical maps and the potential for neuroplasticity are the highest in the acute phase, any delay of the therapy leads to less effective and longer rehabilitation [42,43]. This means that exercise in the early stage following a stroke is important. The methodology presented in this paper is intended for that purpose. Furthermore, the main goal of this study was to devise protocols that enhance the involvement of the patient, thus transforming him/her from mere recipient of physical therapy into the focal point of the therapy protocol. By enabling the patient to be in charge of the intensity and the type of exercise while receiving clear and easy to understand quantitative feedback, such as joint RoM, we hypothesize that the motivation of the patient would also increase.

We have developed seven smart protocols that focus on different movement synergies. There were several reasons why we decided to devise multiple protocols. Firstly, changes in schedule and exercise composition during therapy improve therapy effectiveness [44,45]. Although only two movements were FES-induced (DF and PF of the paretic foot), different activation and deactivation strategies of the stimulation patterns made them variable. Secondly, a variety of motor tasks may possibly increase the motivation of the users and enhance generalization to other motor tasks. Thirdly, one of our goals was to evaluate the effectiveness of individual strategies on joint angles during FES sessions and to examine which protocol was preferred by the patient during our pilot study.

Smart protocols were designed in a manner permitting them to be easily added to the foot drop therapy based on the same FES multi-pad device as: (1) the initial FES protocol in the case of poor balance; (2) the familiarization protocol for patients that never had FES; or (3) the strengthening protocol that cannot endure prolonged walking sessions. It is envisioned that the patient upon reaching sufficient balance and muscle strength necessary for standing and walking, transitions to the assisted walking protocol using the same device. Thus, the familiarization and the building the trust in the device period would be reduced, enabling patients and therapists to fully focus on the gait rehabilitation.

FES-based therapies for foot drop correction mostly apply stimulation to the paretic leg during the gait. It is shown that FES-assisted gait has a significant therapeutic effect on walking ability, gait speed, cadence, symmetry, endurance, and the like, as well as improvement in ankle DF voluntary control [46,47]. Still, stroke survivors need to be able to walk in order to be included in such rehabilitation programs, which can be expected only in the chronic phase of recovery [48]. Hence, introducing new rehabilitation programs for foot drop treatments based on FES which would also cover non-walking patients and employ FES to achieve meaningful functional activation sequences when a patient is sitting or lying, especially in the early stages after the stroke insult, became a topic of research interest. The parameter that could be tracked—given the circumstances and nature of the therapy—is voluntary ankle movement. Ankle control is crucial for walking and it is shown that the supraspinal sensorimotor control of walking can be assessed indirectly by voluntary ankle DF [49].

In our previous FES study for foot drop correction during assisted walking [38] the same system was used as in this study. The time that had passed since the stroke insult of all patients was more than three months and all of them were able to independently walk. The group results showed RoM increase of 14.5° after three weeks of the therapy and an additional 7° in the last week of the therapy. The patient included in the present study did not meet the criteria of independent walking. Her baseline voluntary ankle RoM was smaller than the median RoM value of the walking group (16° compared to 19°), but after three weeks of the therapy, she had a higher increase in the RoM—up to 24°. It should be noted that the ankle movement in seated FES therapy was the only exercise included and ankle joint RoM was the only evaluation metric, thus enabling increased focus of the patient on a specific rehabilitation goal. This could be a possible reason for the higher RoM increase compared to the FES-assisted walking study where all spatio-temporal aspects of gait were of interest.

Sabut et al. [50] observed changes in the voluntary and passive ankle RoMs as the result of a 12-week-long therapy based on FES-assisted walking. The obtained results were compared with the control group. Although the statistically significant increase was present in both RoMs of both groups, the statistically significant difference between groups in terms of the percentage change was found in the voluntary RoM. The mean value of the voluntary RoM of 27 patients (17.3 ± 18.8 months since a stroke) in the FES group increased by 47%, while in our study the patient’s voluntary RoM increased by 150%. The increase of the passive RoM was around 36° in both studies.

Yan et al. sequentially FES-activated four muscles of the leg, mimicking normal gait, while the subjects were lying with the affected leg supported by a sling [51]. The results showed a statistically significant increase in maximum isometric voluntary contraction of ankle dorsiflexors and plantar-flexors in the FES group compared to the placebo and control group. Moreover, the patients that received FES treatment showed a tendency to start walking two to three days earlier than the patients in the other two groups. The findings of this study that focuses on FES exercises initiated shortly after stroke insult, similar to the concept of our study, support the premise that early and intensive intervention could significantly improve motor recovery and functional outcome after a stroke [52].

A novel motor control therapy using CCFES included three stroke survivors in the chronic stage (>6 months since the insult) [53]. The paretic ankle DF was FES-induced with various intensities by the degree of voluntary DF movement of the healthy foot measured with a bend sensor attached to the sock. This protocol is similar to smart protocol 3.1 presented in this paper. One of the outcome measures of six-week home exercises was the difference between the angle of the relaxed ankle in the seated position and the maximal voluntary DF. Two subjects had increased DF angles by 13° and 17°, while the third decreased the DF angle by 2°. The authors presumed that the third patient’s lack of progress could be due to fewer of the prescribed exercises, cognitive deficits, or a depressive state. A larger study failed to demonstrate the advantages of CCFES compared to cyclic FES where patients had a task to synchronously dorsiflex the ankle with the FES [26]. Data from both groups showed significant improvements in the maximum DF angle (median change 6.5°). Besides non-implementing anti-phase ankle movements and the inclusion of patients in the chronic phase, the engagement of patients in both groups was stated as a potential reason for the diminishment of differences. When devising the smart protocols, we took into account all of the above-mentioned factors that contribute to a higher increase in RoM.

Another study that could be qualitatively compared with our methodology, specifically with Smart protocol 1.1, is the mirror therapy combined with FES [54]. The mirror therapy results presented in the paper show statistically significant improvement of ankle RoM compared to conventional therapy.

In this study, except for tracking voluntary and passive RoMs, we focused on FES-induced movements in each smart protocol. Each protocol session was represented with a single angle value. This angle was calculated as the median value of representative FES-induced movements during the protocol (as explained in Results). To compare overall differences between protocols the ANOVA test was used, which didn’t show any significant difference between the protocols (*p* < 0.05). Based on the present finding, it is not possible to divide protocols based on their effectiveness, and thus further research including more patients should be conducted.

In most of the sessions, the smallest value of the median DF FES-induced ankle angle was for protocol 3.2. Reduction in FES-induced DF movement was related to not reaching the maximal stimulation level, which, based on the position of the healthy foot, could be decreased up to 4 mA. This indicates that the patient was often selecting sub-maximal stimulation levels. Nevertheless, despite slightly smaller RoM, there was no statistically significant difference between this and other protocols, suggesting that self-selected stimulation amplitude would have a similar effect as amplitudes that result in maximal stimulated joint movement. This finding also shows the importance of including the patient in the rehabilitation process.

FES-induced DF movements increased during therapy (Figure 6). In the first session mean value of all FES-induced movements was 19°, while in the last it was 33°. Although FES-induced and voluntary PF movements did not increase substantially, the patient succeeded in reliably achieving the minimal threshold for initiating DF (3°) and PF (2°) after eight sessions. For three sessions during, in three sessions the patient did not manage to lower the paretic foot below the minimal threshold (2°).

After the 1st, 6th, 11th, and 15th sessions, the patient chose her preferred protocol. Each time, she chose protocol 1.1 in which feet movement was in-phase. This could be due to in-phase moving of the feet being cognitively easier and more stable than anti-phase moving [55].

Also, we noticed that throughout the first two weeks of the therapy, during protocol 4 (when only the paretic foot should be engaged), she simultaneously symmetrically moved the non-paretic foot. These unintended movements appearing in the contralateral homologous body part during active voluntary moves are called mirror movements [56]. The appearance of post-stroke mirror movements is yet to be fully explained. Some of the possible causes are over activation of the non-lessoned hemisphere [57] or the activity of the phylogenetically-older subcortical motor circuits that contribute to control [58]. In the recent study, it was shown that the disappearance of the mirror movements correlates with the recovery of the affected extremity [59]. We plan on recording these mirror movements in future studies as an additional tool for assessing recovery.

An increase of the passive RoM and a decrease in the gastrocnemius muscle spasticity were noticed in the therapy that combined FES and active participation of the patient in the sense of hand triggering the ankle DF [60]. A similar effect could be extracted from the results presented in this paper. As one can see from Figure 2, there were significant decreases in the passive and/or active RoM on certain days, due to the increased muscle stiffness. As the passive and active RoMs were measured at the beginning of each therapy, the trend of reduced values was not transferred to the RoM (DF+PF) during FES protocols, as seen in Figure 6, indicating that the joint stiffness was alleviated during FES exercises, which is in agreement with the previous study [60,61].

There are several limitations to this study that should be noted. Firstly, as this is a case study, there is uncertainty if the findings on this patient will be confirmed on a larger group of patients. Secondly, the patient was in the acute phase following a stroke insult. Secondly, it is difficult to assess the independent contribution of each parameter—smart protocols, spontaneous recovery, and conventional therapy—to the overall success of the FES therapy. Thirdly, the pillow placed behind the legs was pressing the ankle extensors, resulting in a certain reduction of muscle contractions. Despite that, as the FES protocol was mirroring the standard ankle joint RoM measurement protocol at the Clinic for Rehabilitation of Dr. Miroslav Zotović, the muscle contraction reductions should be similar, meaning that active, passive, and stimulated ROM should also be comparable throughout the study.

The combination of protocols used in each therapy session resulted in increased voluntary and passive RoMs (Figure 2). At the end of the therapy, the patient reached voluntary RoM which corresponds to RoM during normal gait [62]. Better performance of DF and PF movements, as well as an increase of ankle RoM, could be a good base for further gait rehabilitation.

## 5. Conclusions

It is shown that stroke survivors could improve the performance of certain motor skills if they are included in exercises and training [63,64,65,66,67]. We developed a new rehabilitation program that promotes early training of the foot DF and PF. The results from a patient included in this study are promising, but further studies with larger samples are needed to evaluate the utility and effectiveness of this proposed rehabilitation method and to make a distinctions between protocols based on to their contributions to the increase of the ankle joint RoM.

## Figures and Tables

**Table 1:** Smart protocols.

**Protocol 1.1**		
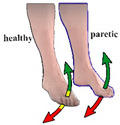	Control	Contralaterally
	Phase	In-phase
	Stimulation activation	Moving above/below positive (10°)/negative (−10°) threshold
	Stimulation termination	Return to the neutral zone
**Protocol 1.2**		
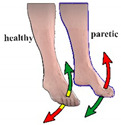	Control	Contralaterally
	Phase	Anti-phase
	Stimulation activation	Moving above/below positive (10°)/negative (−10°) threshold
	Stimulation termination	Return to the neutral zone
**Protocol 2.1**		
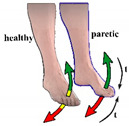	Control	Contralaterally
	Phase	In-phase
	Stimulation activation	Moving foot above/below positive (10°)/negative (−10°) threshold
	Stimulation termination	After a predefined time (3 s). Before subsequent stimulation, the healthy foot must be returned to the neutral zone.
**Protocol 2.2**		
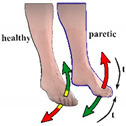	Control	Contralaterally
	Phase	Anti-phase
	Stimulation activation	Moving above/below positive (10°)/negative (−10°) threshold
	Stimulation termination	After a predefined time (3 s). Before subsequent stimulation, the healthy foot must be returned to the neutral zone.
**Protocol 3.1**		
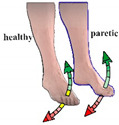	Control	Contralaterally
	Phase	In-phase
	Stimulation activation	The range between an upper threshold (10°) and maximal DF of the healthy foot was divided into five equal levels. The maximal DF and PF angles of the healthy foot were recorded at the beginning of the protocol. In the first level (nearest to the upper threshold), current amplitudes of all pads within DF pad configuration were deducted by 4 mA, in the second by 3 mA, etc. Analogous was for PF movements.
	Stimulation termination	Return to the neutral zone
**Protocol 3.2**		
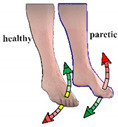	Control	Contralaterally
	Phase	Anti-phase
	Stimulation activation	The range between an upper threshold (10°) and maximal DF of the healthy foot was divided into five equal levels. The maximal DF and PF angles of the healthy foot were recorded at the beginning of the protocol. In the first level (nearest to the upper threshold), current amplitudes of all pads within PF pad configuration were deducted by 4 mA, in the second by 3 mA, etc. Analogous was for PF movements.
	Stimulation termination	Return to the neutral zone
**Protocol 4**		
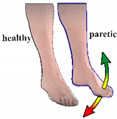	Control	Voluntary movements of the paretic foot
	Stimulation activation	The pad configuration for DF was activated when the foot was moved above the positive threshold (3°) and there was no increase in movement amplitude more than 0.5 s. Analogous was for PF movement, where the threshold was set to 2°.
	Stimulation termination	After a predefined time (3 s)

## Data Availability

Not applicable.

## References

[1] Burridge J., McLellan D. (2000). Relation between abnormal patterns of muscle activation and response to common peroneal nerve stimulation in hemiplegia. J. Neurol. Neurosurg. Psychiatry.

[2] Laufer Y., Ring H., Sprecher E., Hausdorff J.M. (2009). Gait in Individuals with Chronic Hemiparesis: One-Year Follow-up of the Effects of a Neuroprosthesis That Ameliorates Foot Drop. J. Neurol. Phys. Ther..

[3] Taylor P.N., Burridge J.H., Dunkerley A.L., Wood D.E., Norton J.A., Singleton C., Swain I.D. (1999). Clinical use of the odstock dropped foot stimulator: Its effect on the speed and effort of walking. Arch. Phys. Med. Rehabil..

[4] Weber D.J., Stein R.B., Chan K.M., Loeb G.E., Richmond F.J., Rolf R., James K., Chong S.L., Thompson A.K., Misiaszek J. (2004). Functional electrical stimulation using microstimulators to correct foot drop: A case study. Can. J. Physiol. Pharmacol..

[5] Burridge J., Taylor P., Hagan S., Swain I. (1997). Experience of Clinical Use of the Odstock DroppedFoot Stimulator. Artif. Organs.

[6] Stein R.B., Everaert D.G., Thompson A.K., Chong S.L., Whittaker M., Robertson J., Kuether G. (2009). Long-Term Therapeutic and Orthotic Effects of a Foot Drop Stimulator on Walking Performance in Progressive and Nonprogressive Neurological Disorders. Neurorehabilit. Neural Repair.

[7] Stefanovska A., Vodovnik L., Gros N., Rebersek S., Acimovic-Janezic R. (1989). FES and spasticity. IEEE Trans. Biomed. Eng..

[8] Sheffler L.R., Bailey S.N., Wilson R.D., Chae J. (2013). Spatiotemporal, kinematic, and kinetic effects of a peroneal nerve stimulator versus an ankle foot orthosis in hemiparetic gait. Neurorehabilit. Neural Repair.

[9] Ring H., Treger I., Gruendlinger L., Hausdorff J.M. (2009). Neuroprosthesis for Footdrop Compared with an Ankle-Foot Orthosis: Effects on Postural Control during Walking. J. Stroke Cerebrovasc. Dis..

[10] Cozean C., Pease W.S., Hubbell S. (1988). Biofeedback and functional electric stimulation in stroke rehabilitation. Arch. Phys. Med. Rehabil..

[11] Teixeira-Salmela L.F., Nadeau S., Mcbride I., Olney S.J. (2001). Effects of muscle strengthening and physical conditioning training on temporal, kinematic and kinetic variables during gait in chronic stroke survivors. J. Rehabil. Med..

[12] Kerrigan D.C., Karvosky M.E., Riley P.O. (2001). Spastic paretic stiff-legged gait: Joint kinetics. Am. J. Phys. Med. Rehabil..

[13] Peterson C.L., Hall A.L., Kautz S.A., Neptune R.R. (2010). Pre-swing deficits in forward propulsion, swing initiation and power generation by individual muscles during hemiparetic walking. J. Biomech..

[14] Arch E.S., Reisman D.S. (2016). Passive-Dynamic Ankle-Foot Orthoses with Personalized Bending Stiffness Can Enhance Net Plantarflexor Function for Individuals Poststroke. JPO J. Prosthet. Orthot..

[15] Perez M.A., Lungholt B.K.S., Nyborg K., Nielsen J.B. (2004). Motor skill training induces changes in the excitability of the leg cortical area in healthy humans. Exp. Brain Res..

[16] Nudo R.J. (2007). Postinfarct Cortical Plasticity and Behavioral Recovery. Stroke.

[17] Keith R.A. (1997). Treatment strength in rehabilitation. Arch. Phys. Med. Rehabil..

[18] Duncan P.W. (1997). Synthesis of Intervention Trials To Improve Motor Recovery following Stroke. Top. Stroke Rehabil..

[19] Cauraugh J.H., Summers J.J. (2005). Neural plasticity and bilateral movements: A rehabilitation approach for chronic stroke. Prog. Neurobiol..

[20] Burgar C.G., Lum P.S., Shor P.C., Van Der Loos H.F.M. (2001). Development of robots for rehabilitation therapy: The Palo Alto VA/Stanford experience. J. Rehabil. Res. Dev..

[21] Whitall J., Waller S.M., Silver K.H.C., Macko R.F. (2000). Repetitive Bilateral Arm Training with Rhythmic Auditory Cueing Improves Motor Function in Chronic Hemiparetic Stroke. Stroke.

[22] Knutson J.S., Harley M.Y., Hisel T.Z., Chae J. (2007). Improving hand function in stroke survivors: A pilot study of contralaterally controlled functional electric stimulation in chronic hemiplegia. Arch. Phys. Med. Rehabil..

[23] Knutson J.S., Harley M.Y., Hisel T.Z., Hogan S.D., Maloney M.M., Chae J. (2012). Contralaterally controlled functional electrical stimulation for upper extremity hemiplegia: An early-phase randomized clinical trial in subacute stroke patients. Neurorehabil. Neural Repair.

[24] Knutson J.S., Gunzler D.D., Wilson R.D., Chae J. (2016). Contralaterally Controlled Functional Electrical Stimulation Improves Hand Dexterity in Chronic Hemiparesis. Stroke.

[25] Chan M.K.-L., Tong R.K.-Y., Chung K.Y.-K. (2008). Bilateral Upper Limb Training with Functional Electric Stimulation in Patients with Chronic Stroke. Neurorehabilit. Neural Repair.

[26] Knutson J.S., Hansen K., Nagy J., Bailey S.N., Gunzler D.D., Sheffler L.R., Chae J. (2013). Contralaterally controlled neuromuscular electrical stimulation for recovery of ankle dorsiflexion: A pilot randomized controlled trial in chronic stroke patients. Am. J. Phys. Med. Rehabil. Assoc. Acad. Phys..

[27] Kesar T.M., Perumal R., Reisman D.S., Jancosko A., Rudolph K.S., Higginson J.S., Binder-Macleod S.A. (2009). Functional Electrical Stimulation of Ankle Plantarflexor and Dorsiflexor Muscles. Stroke.

[28] Bae S., Lee J., Lee B.-H. (2020). Effect of an EMG–FES Interface on Ankle Joint Training Combined with Real-Time Feedback on Balance and Gait in Patients with Stroke Hemiparesis. Healthcare.

[29] Lotze M., Braun C., Birbaumer N., Anders S., Cohen L.G. (2003). Motor learning elicited by voluntary drive. Brain.

[30] Fugl-Meyer A.R., Jääskö L., Leyman I., Olsson S., Steglind S. (1975). The post-stroke hemiplegic patient. 1. a method for evaluation of physical performance. Scand. J. Rehabil. Med..

[31] Mahoney F.I., Barthel D.W. (1965). Functional evaluation: The Barthel Index: A simple index of independence useful in scoring improvement in the rehabilitation of the chronically ill. Md. State Med. J..

[32] Folstein M.F., Folstein S.E., McHugh P.R. (1975). “Mini-mental state”: A practical method for grading the cognitive state of patients for the clinician. J. Psychiatr. Res..

[33] Malešević J., Malešević N., Bijelić G., Keller T., Konstantinović L. Multi-pad stimulation device for treating foot drop: Case study. Proceedings of the 2014 IEEE 19th International Functional Electrical Stimulation Society Annual Conference (IFESS).

[34] Malešević N.M., Maneski L.Z.P., Ilić V., Jorgovanović N., Bijelić G., Keller T., Popović D.B. (2012). A multi-pad electrode based functional electrical stimulation system for restoration of grasp. J. Neuroeng. Rehabil..

[35] Tan Z., Liu H., Yan T., Jin D., He X., Zheng X., Xu S., Tan C. (2014). The Effectiveness of Functional Electrical Stimulation Based on a Normal Gait Pattern on Subjects with Early Stroke: A Randomized Controlled Trial. BioMed Res. Int..

[36] Rozman J., Sovinec B., Trlep M., Zorko B. (1993). Multielectrode spiral cuff for ordered and reversed activation of nerve fibres. J. Biomed. Eng..

[37] Brurok B., Tãrhaug T., Karlsen T., Leivseth G., Helgerud J., Hoff J. (2013). Effect of lower extremity functional electrical stimulation pulsed isometric contractions on arm cycling peak oxygen uptake in spinal cord injured individuals. J. Rehabil. Med..

[38] Malešević J., Dujović S.D., Savić A.M., Konstantinović L., Vidaković A., Bijelić G., Malešević N., Keller T. (2017). A decision support system for electrode shaping in multi-pad FES foot drop correction. J. Neuroeng. Rehabil..

[39] Peurala S.H., Tarkka I.M., Pitkänen K., Sivenius J. (2005). The Effectiveness of Body Weight-Supported Gait Training and Floor Walking in Patients With Chronic Stroke. Arch. Phys. Med. Rehabil..

[40] Thompson A.K., Stein R.B. (2004). Short-term effects of functional electrical stimulation on motor-evoked potentials in ankle flexor and extensor muscles. Exp. Brain Res..

[41] Tuck K. (2007). Tilt sensing using linear accelerometers. Freescale Semiconductor Application Note AN3107.

[42] Biernaskie J., Chernenko G., Corbett D. (2004). Efficacy of Rehabilitative Experience Declines with Time after Focal Ischemic Brain Injury. J. Neurosci..

[43] Lee R.G., van Donkelaar P. (1995). Mechanisms underlying functional recovery following stroke. Can. J. Neurol. Sci..

[44] Shea C.H., Kohl R.M. (1991). Composition of Practice: Influence on the Retention of Motor Skills. Res. Q. Exerc. Sport.

[45] Hanlon R.E. (1996). Motor learning following unilateral stroke. Arch. Phys. Med. Rehabil..

[46] Sabut S.K., Bhattacharya S.D., Manjunatha M. (2013). Functional Electrical Stimulation on Improving Foot Drop Gait in Poststroke Rehabilitation: A Review of its Technology and Clinical Efficacy. Crit. Rev. Biomed. Eng..

[47] Merletti R., Andina A., Galante M., Furlan I. (1979). Clinical experience of electronic peroneal stimulators in 50 hemiparetic patients. Scand. J. Rehabil. Med..

[48] Kafri M., Laufer Y. (2014). Therapeutic Effects of Functional Electrical Stimulation on Gait in Individuals Post-Stroke. Ann. Biomed. Eng..

[49] Dobkin B.H., Firestine A., West M., Saremi K., Woods R. (2004). Ankle dorsiflexion as an fMRI paradigm to assay motor control for walking during rehabilitation. NeuroImage.

[50] Sabut S.K., Sikdar C., Kumar R., Mahadevappa M. (2011). Functional electrical stimulation of dorsiflexor muscle: Effects on dorsiflexor strength, plantarflexor spasticity, and motor recovery in stroke patients. NeuroRehabilitation.

[51] Yan T., Hui-Chan C.W., Li L.S. (2005). Functional electrical stimulation improves motor recovery of the lower extremity and walking ability of subjects with first acute stroke: A randomized placebo-controlled trial. Stroke.

[52] Kwakkel G., Wagenaar R.C., Koelman T.W., Lankhorst G.J., Koetsier J.C. (1997). Effects of intensity of rehabilitation after stroke: A research synthesis. Stroke.

[53] Knutson J.S., Chae J. (2010). A novel neuromuscular electrical stimulation treatment for recovery of ankle dorsiflexion in chronic hemiplegia: A case series pilot study. Am. J. Phys. Med. Rehabil. Assoc. Acad. Phys..

[54] Salhab G., Sarraj A.R., Saleh S. Mirror therapy combined with functional electrical stimulation for rehabilitation of stroke survivors’ ankle dorsiflexion. Proceedings of the 2016 38th Annual International Conference of the IEEE Engineering in Medicine and Biology Society (EMBC).

[55] Kelso J.A. (1984). Phase transitions and critical behavior in human bimanual coordination. Am. J. Physiol. Integr. Comp. Physiol..

[56] Connolly K., Stratton P. (2008). Developmental Changes in Associated Movements. Dev. Med. Child Neurol..

[57] Cincotta M., Ziemann U. (2008). Neurophysiology of unimanual motor control and mirror movements. Clin. Neurophysiol..

[58] Lawrence D.G., Kuypers H.G. (1968). The functional organization of the motor system in the monkey: II. The effects of lesions of the descending brain-stem pathways. Brain.

[59] Ejaz N., Xu J., Branscheidt M., Hertler B., Schambra H., Widmer M., Faria A.V., Harran M.D., Cortés J.C., Kim N. (2018). Evidence for a subcortical origin of mirror movements after stroke: A longitudinal study. Brain.

[60] Mesci N., Ozdemir F., Kabayel D.D., Tokuc B. (2009). The effects of neuromuscular electrical stimulation on clinical improvement in hemiplegic lower extremity rehabilitation in chronic stroke: A single-blind, randomised, controlled trial. Disabil. Rehabil..

[61] Wang Y.-H., Meng F., Zhang Y., Xu M.-Y., Yue S.-W. (2016). Full-movement neuromuscular electrical stimulation improves plantar flexor spasticity and ankle active dorsiflexion in stroke patients: A randomized controlled study. Clin. Rehabil..

[62] Kadaba M.P., Ramakrishnan H.K., Wootten M.E. (1990). Measurement of lower extremity kinematics during level walking. J. Orthop. Res..

[63] Hesse S., Bertelt C., Jahnke M.T., Schaffrin A., Baake P., Malezic M., Mauritz K.H. (1995). Treadmill Training With Partial Body Weight Support Compared With Physiotherapy in Nonambulatory Hemiparetic Patients. Stroke.

[64] Richards C.L., Malouin F., Wood-Dauphinee S., Williams J., Bouchard J.-P., Brunet D. (1993). Task-specific physical therapy for optimization of gait recovery in acute stroke patients. Arch. Phys. Med. Rehabil..

[65] Yekutiel M., Guttman E. (1993). A controlled trial of the retraining of the sensory function of the hand in stroke patients. J. Neurol. Neurosurg. Psychiatry.

[66] Dean C.M., Shepherd R.B. (1997). Task-Related Training Improves Performance of Seated Reaching Tasks after Stroke. Stroke.

[67] Carr J.H. (1987). Movement Science: Foundations for Physical Therapy in Rehabilitation.

